# Glass transition temperatures of pure glass-forming liquids and binary mixtures

**DOI:** 10.1038/s41598-026-35024-4

**Published:** 2026-01-10

**Authors:** Vitaly Kocherbitov, Ivan Argatov

**Affiliations:** 1 Department of Biomedical Science, Faculty of Health and Society, Malmö University, Malmö, Sweden; 2 Bioflms Research Centre for Biointerfaces, Malmö University, Malmö, Sweden

**Keywords:** Glassy state, Glass transition, Fragility, Relaxation time, Sorption enthalpy, Heat capacity, Activation energy, Chemistry, Materials science, Physics

## Abstract

The glass transition remains one of the most enduring and debated problems in condensed matter science, engaging researchers across disciplines. Two main approaches have traditionally been used to study the glass transition in pure glass formers and their mixtures: the thermodynamic approach and the dynamic (kinetic) approach. The latter focuses on the relaxation dynamics of glasses, with the key parameter being the temperature-dependent relaxation time. Despite decades of research, no physically justified relation between the relaxation time and the glass transition temperature has been established. Here, we propose a new framework for studying the glass transition that builds upon dynamic approaches and bridges them with thermodynamic concepts. We derive equations for the glass transition temperature based on relaxation parameters and demonstrate that the relaxation time at the glass transition is not constant—as is commonly assumed on the laboratory timescale—but depends on the intrinsic relaxation characteristics of the system. Extending this framework to mixtures of glass formers, we show that the Gordon–Taylor equation naturally emerges from ideal dynamic mixing rules. However, for accurate prediction of glass transition properties in mixtures, understanding deviations from ideal mixing remains crucial.

**Supplementary Information:**

The online version contains supplementary material available at 10.1038/s41598-026-35024-4.

## Introduction

The glass transition is one of the most enduring and debated problems in condensed matter science, engaging researchers across physics, chemistry, materials science, and engineering^1,2^. Unlike crystalline melting, which occurs at a sharp equilibrium temperature, vitrification is a kinetic phenomenon characterized by the dramatic slowdown of molecular motion upon cooling. At the glass transition temperature ${T}_{\text{g}}$, the structural relaxation time becomes so large that the liquid falls out of equilibrium on experimental time scales, transforming into a disordered amorphous solid. Being cooling rate dependent, this transformation is not a conventional first—or second-order thermodynamic transition, yet it exhibits clear dynamic and thermodynamic signatures that define the field of glass transition research.

A key dynamic parameter is the structural α-relaxation time $\tau$, describing cooperative molecular rearrangements^3^. Near ${T}_{\text{g}}$, relaxation time can increase by many orders of magnitude within a narrow temperature range. In relaxation studies, the glass transition temperature is operationally defined as the temperature at which the α-relaxation time of a supercooled liquid reaches a conventional timescale of about 100 or 1000 s^4,5^. Despite its practicality, this definition lacks an explicit link to the rate of temperature change, which limits its use, especially in experiments with extreme heating or cooling rates.

**Fig. 1:**
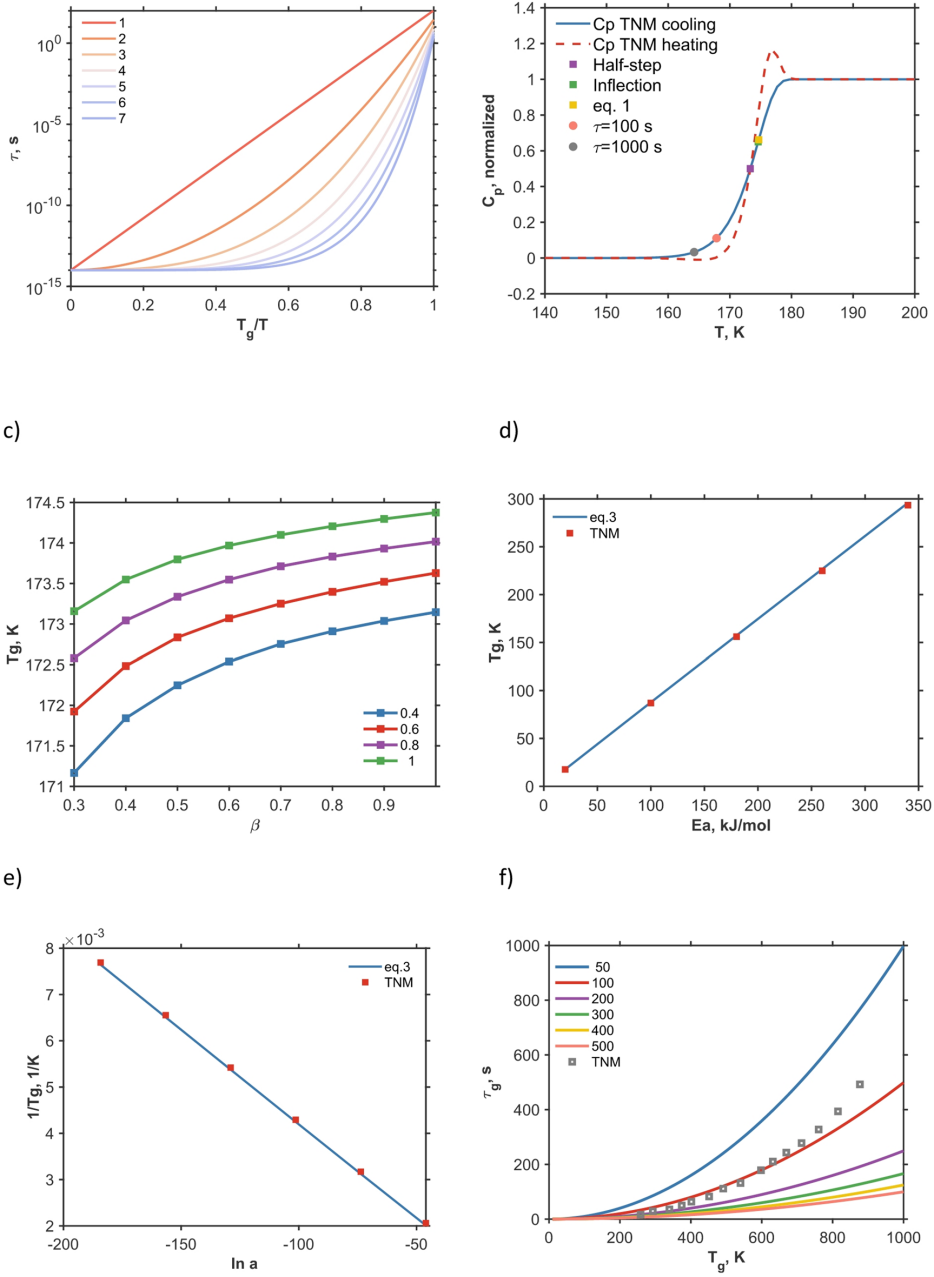
Illustration of glass transition properties and the approach presented here. A schematic illustration of the relaxation time for different values of $\alpha$ parameter of AM model in Angell coordinates (**a**). The heat capacity in a cooling-heating scan generated using Tool—Narayanaswami—Moynihan (TNM) model, the points show ${T}_{\text{g}}$ determined on cooling using five different methods (**b**). The dependence of ${T}_{\text{g}}$ on $\beta$ and $x$ parameters in TNM model (**c**). The dependence of ${T}_{\text{g}}$ on activation energy for Eq. 3 and TNM model (**d**) and the dependence of reciprocal ${T}_{\text{g}}$ on $\text{ln}a$ for the two cases. For TNM model, when fixed, the parameter values are: $a$=2·10^–60^, ${E}_{a}$=200 kJ/mol, $\beta$=0.6, $x$=0.7. (**e**). Variation of relaxation time at the glass transition as dependent on the glass transition temperature calculated using Eq. 7; the legend shows activation energies in kJ/mol, the scan rate is 10 K/min, for TNM model ${E}_{a}$=200 kJ/mol and ${T}_{\text{g}}$ was determined as an inflection point (**f**).

In simple activated processes, the temperature dependence of relaxation time can be described by the Arrhenius equation, in which the dynamics is governed by a constant activation energy ${E}_{a}$. However, most glass formers strongly deviate from the Arrhenius behavior. Instead, they exhibit a temperature-dependent activation energy that increases as ${T}_{\text{g}}$ is approached. To quantify and classify these deviations, Angell introduced the concept of fragility^2^ which can be conveniently illustrated using the Angell plot. In this representation, relaxation time or viscosity is plotted against the reduced inverse temperature ${T}_{\text{g}}/T$ (Fig. 1.a). Strong liquids (e.g., SiO₂) display nearly Arrhenius-like behavior with an almost constant activation energy, while fragile liquids (e.g., o-terphenyl^6^) show a steep, super-Arrhenius increase corresponding to rapidly growing apparent activation energies. Fragility has since become a central concept linking the kinetic slowdown of supercooled liquids with underlying structural and thermodynamic characteristics^7^.

Dynamic responses of supercooled liquids close to ${T}_{\text{g}}$ are non-exponential: relaxation functions typically follow the stretched exponential Kohlrausch–Williams–Watts (KWW) form^1^, reflecting the distribution of local relaxation times and structural heterogeneity. To describe structural relaxation under nonequilibrium conditions, phenomenological frameworks such as the Tool–Narayanaswamy–Moynihan (TNM) model^8^ are widely employed. These models capture memory effects and structural recovery, enabling quantitative interpretation of calorimetric data on glass transitions^9^. Beyond phenomenological approaches, theoretical frameworks such as Mode Coupling Theory (MCT) have been developed to describe the rapid slowdown of dynamics in supercooled liquids^10^. MCT predicts a power-law divergence of relaxation times at a critical temperature ${{T}_{c}>T}_{\text{g}}$, capturing key features of the early stages of dynamic arrest. Another powerful conceptual approach is the energy landscape perspective, in which the dynamics of supercooled liquids are mapped onto the topography of a multidimensional potential energy surface [19]. In this view, the slowing down of dynamics is attributed to the system’s progressive confinement in deeper basins of the landscape^1^, with structural relaxation involving collective rearrangements between minima.

Molecular dynamics (MD) simulations have become an indispensable tool for investigating the glass transition in supercooled liquids^11–14^. By modeling the trajectories of atoms and molecules with classical force fields, MD provides access to atomic-scale dynamics near the glass transition temperature and yields insights into relaxation processes at the molecular level. This approach is particularly valuable for systems prone to crystallization, such as pure water, where large portions of the supercooled regime remain experimentally inaccessible. A major limitation, however, arises from the substantial discrepancy between the cooling rates typically employed in MD simulations (10^9^ K/s) and those achievable in experiments (10^0^ K/s). Consequently, understanding the influence of cooling rate—especially in connection with activation energy and fragility concepts—is of critical importance. Recently, deep learning and artificial intelligence–based techniques have emerged as powerful complements to traditional computational methods in the study of glass-related phenomena^15–17^.

Despite not being a true equilibrium thermodynamic transition, the glass transition is often treated as second order–like, characterized by a discontinuity in the slope of enthalpy, experimentally observed as a change in heat capacity, $\Delta {C}_{p}$ at ${T}_{\text{g}}$, reflecting the freezing of configurational degrees of freedom. In this framework, ${T}_{\text{g}}$ is operationally defined as either the midpoint or the inflection point of the heat-capacity step. In multicomponent systems, the dependence of ${T}_{\text{g}}$ on composition is often described by empirical relations such as the Gordon–Taylor Eq.^18^ containing an empirical constant $\kappa$. It was suggested^19,20^ that the Gordon–Taylor parameter $\kappa$ reflects the ratio of the heat capacity increments of the two components $\Delta {C}_{p2}^{0}/\Delta {C}_{p1}^{0}$, but the theoretical justification for this relation is not rigorous. Nonetheless, when $\kappa$ is used as an empirical parameter, the Gordon-Taylor equation successfully describes the ${T}_{\text{g}}$ of polymer blends and amorphous solid dispersions. To the best of our knowledge, no interpretation of this parameter from the kinetic or dynamics perspectives has been proposed. The Gordon–Taylor equation remains of great relevance in polymer science, food technology, and pharmaceuticals, where compositional tuning of ${T}_{\text{g}}$ is crucial for product design and stability^21,22^. Overall, the composition dependence of the glass transition temperature in mixtures remains an important unsolved problem^23^.

In this work, we propose an analytical expression for the glass transition temperature of a glass-forming liquid based on its relaxation parameters. This approach uncovers an unexpected link between the relaxation time at the glass transition, ${\tau }_{\text{g}}$, and the activation energy, and establishes a rigorous definition applicable across different rates of temperature change. Furthermore, we demonstrate that the ${T}_{\text{g}}$ of a mixture can be expressed in terms of the relaxation parameters of its components, and that the Gordon–Taylor equation emerges as a particular case of the proposed framework. Finally, we discuss the implications of dynamic mixing rules for the determination of glass transition temperatures.

### Glass transition temperature in glass-forming liquids: relation to relaxation parameters.

There are several models expressing the dependence of the relaxation time $\tau$ on temperature, including Arrhenius, Vogel–Fulcher–Tammann (VFT), Avramov–Milchev (AM)^24^, Waterton–Mauro (WM)^25^, Drozd-Rzoska^26,27^ models, some of these are presented in Table 1. Unfortunately, the glass transition temperature ${T}_{\text{g}}$ cannot be directly evaluated from these models. A practical option is to use the 100- or 1000-s criteria mentioned in the introduction; however, this approach does not explicitly account for the essential feature of the glass transition—its dynamic nature, since ${T}_{\text{g}}$ depends on the temperature change rate $q$. Existing approaches describing the glass transition such as TNM^8^, KAHR^28^, Minimal Model (MM)^29^ focus on calculation of other parameters, for example the heat capacity *C*_*p*_ or analogous volumetric data as a function of temperature. Then using certain criteria such as half-step or inflection point, one can calculate ${T}_{\text{g}}$ . Although in the simplest case of the Minimal Model (MM) an approximate analytical solution has been proposed^29^, a general solution to the problem has not been presented before.

In this work we explore a different approach, based on the equation proposed by Volkenshtein and Ptitsyn^30^, that relates the cooling or heating rate $q=dT/dt$ and the temperature dependence of the relaxation time:

1 $$\left( {\frac{d\tau }{{dT}}} \right)_{{T = T_{{\text{g}}} }} = - \frac{1}{\left| q \right|}{ }.$$

This equation can be justified as follows: in the liquid state, where the relaxation time is small, its change $\Delta \tau$ is smaller than the time $\Delta t$ allowed for a temperature change $\Delta T$. In the glassy state relaxation becomes slow, the structure cannot equilibrate on the experimental time scale, and the system falls out of equilibrium. In this regime $\Delta \tau$ is larger than $\Delta t$.The point where these times coincide ($\Delta \tau$ =$\Delta t$) defines the glass transition temperature. Then, expressing $\Delta t$ through the scan rate $q$ one arrives at Eq. 1. Using this approach, transcendental equations for ${T}_{\text{g}}$ can be readily derived by differentiating the expressions for $\tau$ corresponding to the models listed in Table 1. For the simplest case of Arrhenius dependence, it reads:

2 $$\frac{ab}{{T_{{\text{g}}}^{2} }}\exp \left( {\frac{b}{{T_{{\text{g}}} }}} \right) = 1,$$

where the parameters are defined as $a=q{\tau }_{0}$ and $b={E}_{a}/R$. The parameter ${\tau }_{0}$ represents an extrapolation of the relaxation time to infinite temperatures and for Arrhenius model it does not have a clear physical meaning for majority of glass formers, while for models describing non-Arrhenius behavior it is usually assumed to be close to 10^–13^ or 10^–14^ s. Expressions analogous to Eq. 2 for other models are presented in SI. Although this equation cannot be directly used for ${T}_{\text{g}}$ calculation, it can be solved using Lambert W function $y={W}_{0}(x)$ where $y \text{exp}\left(y\right)=x$. This function was introduced few decades ago^31^ and relatively recently became available in widely used software packages. There are several examples of applications of Lambert W function for glass transition studies in literature^32,33^, but to the best of our knowledge it has not been applied for calculation of the glass transition temperature. Expressions for glass transition temperatures for three common relaxation times models are presented in Table 1, details of their derivations are shown in SI. These equations allow calculations of ${T}_{\text{g}}$ when relaxation models parameters are known. Alternatively, they can also be used to calculate parameters of relaxation models (for example ${\tau }_{0}$) combining the ${T}_{\text{g}}$ value with a partial information on the relaxation properties, obtained *e.g.,* from scan rate dependence of ${T}_{\text{g}}$.

Two out of three models presented in Table 1 can describe the non-Arrhenius relaxation behavior and hence can describe the global evolution of relaxation time and activation energy in a broad temperature range. Apart from this behavior, there is also non-linearity and non-exponentiality phenomena that are most relevant in the glass transition region. The effect of these phenomena can be discussed using the Tool—Narayanaswami—Moynihan (TNM) model^8,34,35^, see Fig. 1. The non-linearity and non-exponentiality strongly affect the shape of the heat capacity curve in the glass transition region and influence the width of the glass transition^9^. Nonetheless, the effect of the non-exponentiality and non-linearity parameters (denoted as $\beta$ and *x* respectively) on ${T}_{\text{g}}$ is rather limited, as illustrated in Fig. 1.c, and can be disregarded keeping in mind typical accuracies of glass transition temperature measurements. Moreover, although for understanding the global temperature dependence of relaxation times, non-Arrhenius behavior expressed by VFT and AM models is crucial, variation of activation energy inside the glass transition region in terms of these models will be minor. Hence, the performance of the proposed approach can be illustrated using the heat capacity data generated by TNM model. Note that when parameters $\beta$ and *x* equal one, TNM model is identical to the minimal model (MM)^29^.

**Table 1:** Relaxation times models and corresponding expressions for glass transition temperatures.

Model	Relaxation time, $\:\tau\:$	Activation energy, $\:{E}_{a}\left(T\right)/R$	Glass transition temperature, $\:{T}_{\text{g}}$	
Arrhenius	$\:{\tau\:}_{0}\text{e}\text{x}\text{p}\left\{\frac{b}{T}\right\}$	$\:b$	$\:\frac{b}{2{W}_{0}\left(\frac{1}{2}\sqrt{\frac{b}{a}}\right)}$	(3)
Vogel–Fulcher–Tammann (VFT)	$\:{\tau\:}_{0}\text{e}\text{x}\text{p}\left\{\frac{B}{T-{T}_{0}}\right\}$	$\:B{\left(1-\frac{{T}_{0}}{T}\right)}^{-2}$	$\:{T}_{0}+\frac{B}{2{W}_{0}\left(\frac{1}{2}\sqrt{\frac{B}{a}}\right)}$	(4)
Avramov–Milchev (AM)	$\:{\tau\:}_{0}\text{e}\text{x}\text{p}\left\{{\left(\frac{\mathcal{B}}{T}\right)}^{\alpha\:}\right\}$	$\:{\alpha\:\mathcal{B}\left(\frac{\mathcal{B}}{T}\right)}^{\alpha\:-1}$	$\:\frac{{b}_{\text{g}}}{{\alpha\:W}_{0}\left(\frac{1}{2\alpha\:}\sqrt{\frac{{b}_{\text{g}}}{a}}\right)},\:\:{b}_{\text{g}}=\frac{{E}_{a}\left({T}_{\text{g}}\right)}{R}$	(5)

Figure 1b illustrates applicability of Eq. 1 for determining the glass transition temperature. This equation offers an alternative definition of the glass transition temperature in addition to half-step and inflection point methods well-established in literature. Although it does strictly coincide with either of the ${T}_{\text{g}}$ values determined using traditional methods, its deviation from the inflection point value is less than 0.4 K in this example. Being less practical for the treatment of heat capacity data, this method has an advantage of a clearer definition of the glass transition point from the relaxation phenomena perspective.

Equation 3 reveals a relationship between the relaxation parameters ${\tau }_{0}$ and ${E}_{a}$ and the glass transition temperature, as illustrated in Fig. 1.d-e. Because this relationship pertains exclusively to the glass transition temperature, activation energy variations at higher temperatures are not relevant. Consequently, Eq. 3 and Fig. 1.d-e are applicable for other models, provided that ${\tau }_{0}$ and ${E}_{a}$ are evaluated at ${T}_{\text{g}}$. Despite the complex form of Eq. 3, the dependences of ${T}_{\text{g}}$ on ${E}_{a}$ and ${1/T}_{\text{g}}$ on $\text{ln}a$ are very close to linear and also in good agreement with TNM model results (shown as squares in the figure).

The relation between ${T}_{\text{g}}$ and the scan rate *q* can be analyzed based on Eq. 2. By writing the expression for $\text{ln}q$ and differentiating it with respect to ${1/T}_{\text{g}}$, we arrive at the following expression:

6 $$\frac{d\ln \left| q \right|}{{d\left( {1/T_{{\text{g}}} } \right)}} = - b - 2T_{{\text{g}}}$$

This expression has an additional term compared with the well-known Moynihan Eq. ^34,36^. Although this term is smaller than $b$, its presence can affect the accuracy of the activation energy assessment based on the scan-rate dependence of ${T}_{\text{g}}$. For example, activation energy values obtained using Moynihan-equation treatment of generated heat capacity data^9^ were slightly higher than the values used to generate those data. Equation 6 readily explains this discrepancy.

There is an additional consequence of using Eq. 1, which defines the derivative of relaxation time with respect to temperature at ${T}_{\text{g}}$. Usually in literature the relaxation time at the glass transition is assumed to be either 100 or 1000 s, i.e., characteristic time of the laboratory time scale. Despite being often used, this assumption is rather arbitrary and only roughly corresponds to typical experimental or “laboratory” time scale. However, since glass is a thermodynamically nonequilibrium nonstable state of matter^37^, (with a possible exception of vapor-deposited glasses^38,39^ that are claimed to be more stable) explicit consideration of the scan rate $q$ is crucial for understanding the dynamical nature of the glass transition. As illustrated in Fig. 1.b, employing these relaxation time values would lead to a substantial underestimation of the glass transition temperature. Moreover, systems with different activation energies will respond differently to temperature changes (see equations for $\tau$ in Table 1), which implies different interplay with the temperature scanning expressed by the scan rate $q$. Combining the Arrhenius behavior of $\tau$ with Eq. 2, one arrives at the following equation:

7 $$\tau_{{\text{g}}} = \frac{{T_{{\text{g}}}^{2} }}{{\left| q \right|b_{{\text{g}}} }} .$$

This equation shows that the relaxation time at the glass transition is not constant but can vary depending not only on scan rate $q$, but also on ${T}_{\text{g}}$ and activation energy. In particular, at constant activation energy ${\tau }_{\text{g}}$ is higher for higher ${T}_{\text{g}}$ values, while at constant ${T}_{\text{g}}$ it is reversely proportional to activation energy. Notably, this variation can be very substantial – up to three orders of magnitude according to the example in Fig. 1.f. It is important to stress that this variation is not merely a consequence of the definition of ${T}_{\text{g}}$ used here (Eq. 1). Figure 1.f also shows dependence of ${\tau }_{\text{g}}$ on ${T}_{\text{g}}$ calculated the inflection point in simulated TNM cooling scans. Although due to different formal definitions of ${T}_{\text{g}}$, there is certain quantitative difference between the TNM data and results of Eq. 7 at high temperatures, the general trend is consistent.

In summary, the results presented in this section provide a clear framework for calculating the temperature and relaxation time of glass transition from the parameters of relaxation models. Additional results for other relaxation models are presented in Supporting Information.

### Glass transition in binary mixtures

#### Thermodynamic vs dynamic approach

Another problem that often arises in glass transition topic is calculation of the glass transition temperature of a mixture using glass transition temperatures of pure components. The ${T}_{\text{g}}$ of binary mixtures is usually calculated using the Gordon-Taylor equation (Eq. 1), where $\kappa$ is a fitting parameter. Although there are publications based on classical thermodynamic approach^19,40^ claiming that this parameter is identical to the heat capacity increments ratio $\Delta {c}_{p2}^{0}/\Delta {c}_{p1}^{0}$, the justification of this equality is based on extra-thermodynamic assumptions. A rigorous treatment of the thermal cycle leads to more complex dependence. From a rigorous thermodynamic model^41^ that describes the effect of glass transition on the enthalpy of sorption, it follows that

8 $$\kappa \approx \frac{{{\Delta }c_{p2}^{0} }}{{{\Delta }c_{p1}^{0} }}\left( {\frac{{T_{{{\text{g}}2}} - T}}{{T_{{{\text{g}}2}} - T_{{{\text{g}}1}} }} - \frac{{{\Delta }H_{1}^{{{\text{m}}0}} \left( T \right)}}{{M_{1} \left( {T_{{{\text{g}}2}} - T_{{{\text{g}}1}} } \right){\Delta }c_{p1}^{0} }}} \right)^{ - 1} ,$$

where $\Delta {H}_{1}^{\text{m}0}$ is the enthalpy increment in the zero solute concentration limit, $T$ is a temperature at which $\Delta {H}_{1}^{\text{m}0}$ is measured, and ${M}_{1}$ is the molar mass of the solute. It is easy to see that the second factor on the right-hand side of Eq. (7) can significantly differ from one, as for instance, it equals to $0.497$ in the case ${M}_{1}=18.015$ g/mol, ${T}_{\text{g}1}=137$ K, ${T}_{\text{g}2}=473$ K, $\Delta {c}_{p1}^{0}=2$ J/gK, and $T=298$ K, $\Delta {H}_{1}^{\text{m}0}\left(T\right)=-18$ kJ/mol (the value typically observed for carbohydrate polymers^42,43^).

In this section, we present an alternative method for deriving the composition dependence of the glass transition temperature, based on a dynamic rather than a thermodynamic approach, emphasizing the non-equilibrium nature of the glass transition. In this framework, the Gordon–Taylor equation is obtained as an asymptotic approximation of more general approach based on dynamic mixing rules.

### Dynamic mixing rules

For clarity, we start with the simplest case when both temperature and composition dependences are described by Arrhenius expressions and then generalize it for other cases. We assume that Eq. (3) applies for both components 1 and 2 of the mixture but with different model parameters: ${a}_{1}$, ${b}_{1}$ and ${a}_{2}$, ${b}_{2}$ respectively. Analogously to the Arrhenius mixing rule for viscosity $\text{ln}\eta ={w}_{1}\text{ln}{\eta }_{1}+{w}_{2}\text{ln}{\eta }_{2}$ we formulate the ideal dynamic mixing (IDM) rules as follows:

9 $$b = w_{1} b_{1} + w_{2} b_{2} , \ln \frac{a}{b} = w_{1} \ln \frac{{a_{1} }}{{b_{1} }} + w_{2} \ln \frac{{a_{2} }}{{b_{2} }}.$$

It should be emphasized that both primary model parameters a and b have the physical dimension of temperature, and therefore the logarithms in the second formula (8) are taken of dimensionless quantities. It is easy to show (see Supporting Info) that the logarithmic mixing rule for the a/b ratio is approximately equivalent to the linear mixing rule of fragilities: $m = w_{1} m_{1} + w_{2} m_{2}$ . We note also that the logarithmic rules of additivity similar to (9) were previously used in a number of studies, e.g., for estimating viscosity^44–46^ and Arrhenius activation energy^47^. Here the word “dynamic” highlights that IDM does not imply ideal mixing in thermodynamic sense but rather address dynamic and kinetic properties of the system.

The substitution of the ideal dynamic mixing rules (9) to Eq. 3 and making use of the ${T}_{\text{g}}$ expressions for pure components (see details in SI), yields an equation for glass transition temperature of a binary mixture:

10 $$T_{{\text{g}}} = \frac{{w_{1} T_{{{\text{g}}1}} W_{0} \left( {\Lambda_{1} } \right) + w_{2} T_{{{\text{g}}2}} W_{0} \left( {\Lambda_{2} } \right)}}{{W_{0} \left( {\Lambda_{1}^{{w_{1} }} \Lambda_{2}^{{w_{2} }} } \right)}}.$$

Here we have introduced the notation hasized that both prim. We note that in the limiting cases when either ${w}_{1}=0$ or ${w}_{2}=0$, the above formula results in ${T}_{\text{g}}={T}_{\text{g}2}$ or ${T}_{\text{g}}={T}_{\text{g}1}$, respectively. In these terms, ${T}_{\text{g}}$ and $\Lambda$ are related as shown below:

11 $$T_{g} = \frac{b}{{2W_{0} \left( \Lambda \right)}},\Lambda = \frac{b}{{2T_{g} }}\exp \left( {\frac{b}{{2T_{{\text{g}}} }}} \right).$$

For the cases when ${\Lambda }_{i}$ values are large, assuming ${W}_{0}\left({\Lambda }_{2}\right)\approx \text{ln}{(\Lambda }_{2})$, Eq. 10 can be further modified:

12 $$T_{{\text{g}}} = \frac{{w_{1} T_{{{\text{g}}1}} \ln \left( {\Lambda_{1} } \right) + w_{2} T_{{{\text{g}}2}} \ln \left( {\Lambda_{2} } \right)}}{{w_{1} \ln \left( {\Lambda_{1} } \right) + w_{2} \ln \left( {\Lambda_{2} } \right)}}.$$

It is easy to see that dividing both nominator and denominator in Eq. 12 by $\text{ln}\left({\Lambda }_{1}\right)$, one recovers the Gordon-Taylor equation

13 $$T_{{\text{g}}} = \frac{{w_{1} T_{{{\text{g}}1}} + \kappa w_{2} T_{{{\text{g}}2}} }}{{w_{1} + \kappa w_{2} }},$$

and the $\kappa$ parameter can be defined using ratios of activation energies or fragilities: $\kappa =\frac{\text{ln}({\Lambda }_{2})}{\text{ln}({\Lambda }_{1})}\approx \frac{{E}_{a2}{T}_{\text{g}1}}{{E}_{a1}{T}_{\text{g}2}}=\frac{{m}_{2}}{{m}_{1}}$. Here the approximate equality is valid for substantially large ${\Lambda }_{1}$, ${\Lambda }_{2}$ values and ${m}_{1}$, ${m}_{2}$ are fragilities of the components. Interestingly, the Gordon-Taylor equation can be derived directly using the fragility concept, see Sect.  2.2.2 in supporting info. We suggest, however, that derivation using parameters *a* and *b* is more fundamental since it only uses relaxation parameters, while definition of fragility contains the ${T}_{\text{g}}$ value.

While in Eqs. 10 and 12 the glass transition temperature of a mixture is defined by the properties of its individual components—namely, their glass transition temperatures and activation energies (or, equivalently, fragilities)—these equations assume ideal dynamic mixing (IDM), an assumption that is not always valid. Consequently, the above expression for κ may not be accurate under IDM, just as its interpretation as the ratio of heat capacity increments is not strictly valid in the thermodynamic approach.

**Fig. 2:**
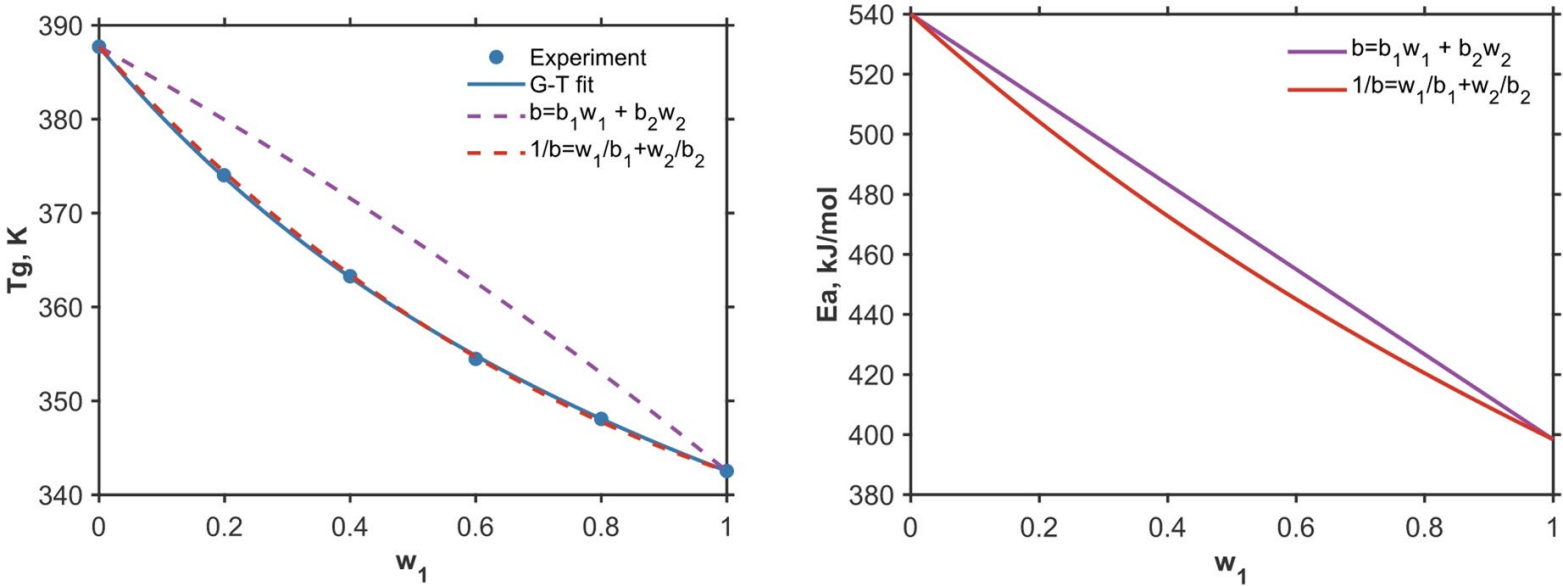
The glass transition in the system sucrose-trehalose, the experimental data are from ref^48^, the dashed curves are calculated using two different mixing rules (**a**). The corresponding activation energy of the mixture. The ${E}_{a}$ values for the pure components are assumed to be 398 and 540 kJ/mol respectively (**b**).

An example show in Fig. 2 illustrates the glass transition temperature in the practically important sucrose-trehalose system. Despite having the same chemical formula, these two carbohydrates exhibit substantially different glass transition temperatures and activation energies. The slight deviation from the linearity in the concentration dependence of the glass transition^48^ (Fig. 2a) correlates with a subtle negative deviation from the linearity in activation energy (Fig. 2b), which can be described by the harmonic mixing rule $1/b={w}_{1}/{b}_{1}+{w}_{2}/{b}_{2}$. More complex concentration dependences of ${T}_{\text{g}}$ and ${E}_{a}$ for more dissimilar components are discussed in the next section.

### Non-Arrhenius behavior in binary systems

The glass transition temperature of a binary mixture for non-Arrhenius relaxation time temperature dependence is best illustrated using Avramov–Milchev model (Eq. 5). Although for accurate description of experimental data on relaxation time or viscosity all three parameters of the model can be used as free fitting parameters, in terms of Angell plot (Fig. 1a) it is convenient to assume $a=q{\tau }_{0}$ being a constant. Typical values of ${\tau }_{0}$ constant used in literature are 10^–13^ and 10^–14^ s and correspond to segmental relaxation time in the high temperature limit^49^. In this case AM model becomes a two-parameter model, i.e. only mixing rules for $\alpha$ and ${b}_{g}$ (or $B$) are needed for calculation of ${T}_{\text{g}}$ using the equation ${T}_{\text{g}}={b}_{\text{g}}/2\alpha {W}_{0}\left(\frac{1}{2\alpha }\sqrt{\frac{{b}_{\text{g}}}{a}}\right)$ (see also Eq. S38).

In case if glass transition temperatures of pure components are known, only one degree of freedom remains, for example if the parameter ${\alpha }_{1}$ and its mixing rules are defined, the activation energy for component 1 can be calculated as follows: ${b}_{\text{g}1}={\alpha }_{1}{T}_{\text{g}1}{W}_{0}\left(\frac{{T}_{\text{g}1}}{{\alpha }_{1}a}\right)$, and analogously for the second component.

Introducing non-ideal dynamic mixing (NDM) rules for AM parameters, which cover both linear ($n=1$) and non-linear ($n \ne 1$) composition dependencies:

14 $$\alpha = \left( {1 - w_{2}^{n} } \right)\alpha_{1} + w_{2}^{n} \alpha_{2} ,\;b_{g} = \left( {1 - w_{2}^{n} } \right)b_{g1}$$

one can directly calculate the glass transition temperature using $\alpha$ and ${b}_{\text{g}}$ values for the mixture using Eq. 5. An explicit formula in terms of parameters for pure components is shown in SI.

**Fig. 3:**
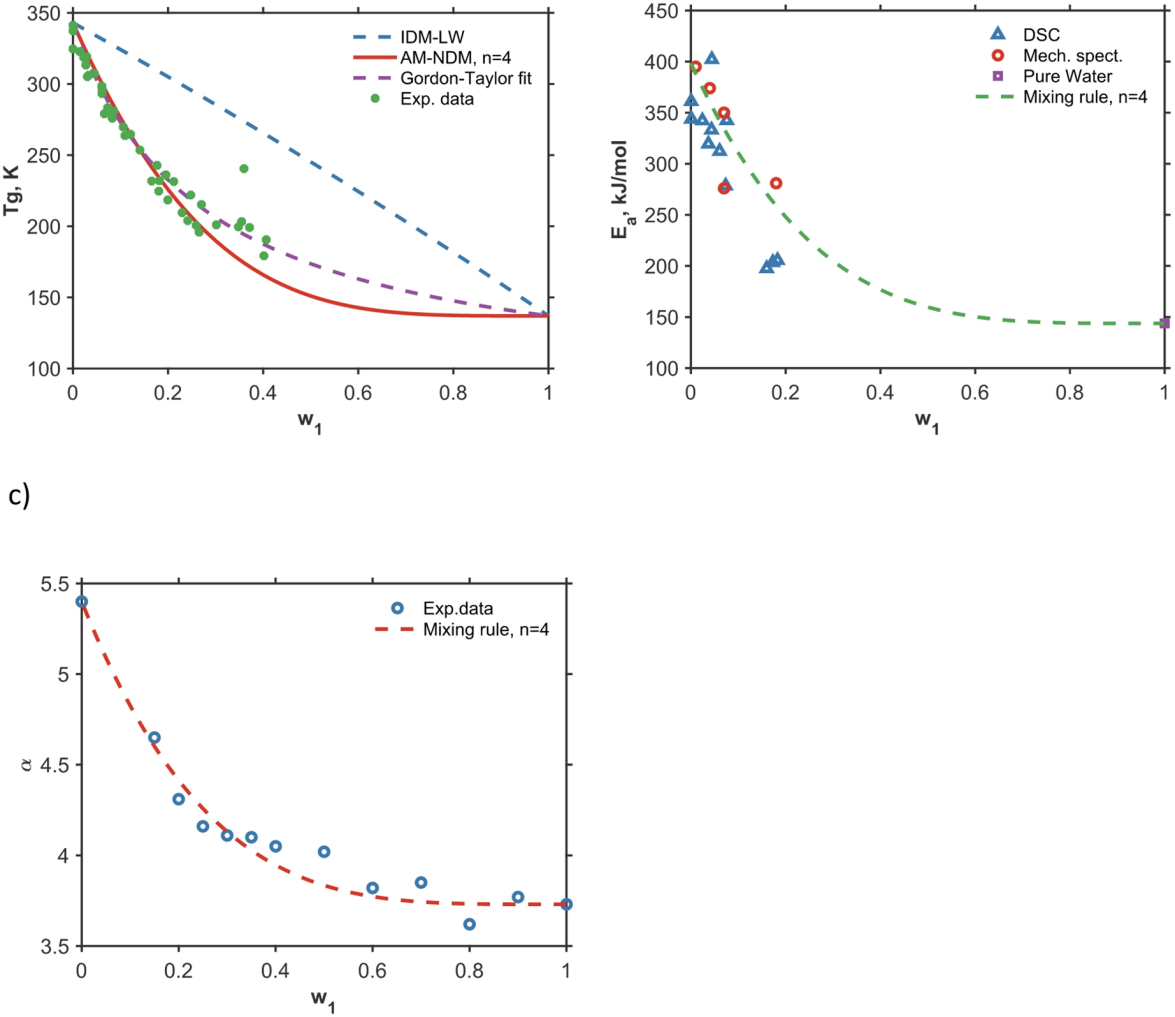
The glass transition temperature in the water-sucrose system: the circles are experimental data taken from ref^50^; AM-NDM denotes calculation using Eqs. 5, 13; Gordon-Taylor fit (purple dashed curve) is calculated from AM-NDM data only for water contents up to 0.2 (**a**). Activation energy in the same system, experimental data from ref^9^ (**b**). AM model parameter $\alpha$ in the system water-sucrose, experimental data are from ref^51^ (**c**).

To illustrate the importance of non-Arrhenius behavior and non-ideal dynamic mixing rules, we chose the binary sucrose–water system, which is highly relevant for pharmaceutical, biomedical, and food applications. Calculation of ${T}_{\text{g}}$ using IDM (blue curve in Fig. 3a) clearly does not provide a reasonable result: the curve is a negative second derivative, clearly contradicting well-known experimental behavior. In terms of Eq. 12 and 13 it can be explained by the fact that the activation energy of sucrose is higher than that of water, which formally results in Gordon-Taylor coefficient higher than one. To find the reason for this discrepancy, it is instructive to analyze the data on concentration dependence of activation energy and alpha parameter in this system (Fig. 3b, c). Clearly, according to literature data, these parameters exhibit a non-linear behavior which can be well described by Eq. 14 using *n* = 4. Using this mixing rule, the glass transition in sucrose-water system can be predicted in a broad concentration range.

From a molecular perspective, this result shows that addition of limited amount of water to dehydrated carbohydrate decreases the activation energy of the glass transition stronger than it could be expected from the linear combination of the components’ properties.

## Conclusions

In this work, we introduced a unified framework for calculating the glass transition temperature in both pure glass formers and their mixtures. Grounded in kinetic considerations of relaxation processes, the framework naturally connects to phenomenological and thermodynamic descriptions of the glass transition. The central result is the derivation of explicit equations for the glass transition temperature, expressed in terms of relaxation parameters, using the Lambert W function. This formalism demonstrates that the relaxation time at the glass transition is not universal—as often assumed on the laboratory timescale—but instead depends on the intrinsic relaxation characteristics of the system. Such an approach is particularly useful for describing glass transitions under nonstandard conditions, such as measurements at unusual scan rates or molecular dynamics simulations.

Extending the framework to mixtures, we demonstrated that the Gordon–Taylor equation naturally arises from ideal dynamic mixing rules, but for real systems different mixing rules can be more adequate. To test the applicability of the approach, we analyzed experimental data on binary mixtures of fragile glass formers sucrose-trehalose and sucrose–water and compared with the theoretical predictions.

Overall, the proposed framework provides a consistent theoretical basis for description of the glass transition temperatures in both pure glass formers and mixtures, offering new opportunities for quantitative modeling of complex glass-forming systems.

## Supplementary Information

Below is the link to the electronic supplementary material.

Supplementary Material 1

## Data Availability

The datasets used and/or analysed during the current study available from the corresponding author on reasonable request.
