# Supplementary material for: Glass transition temperatures of pure glass-forming liquids and binary mixtures
Source: Sci Rep. 2026 Jan 10;16:1317. doi: 10.1038/s41598-026-35024-4 (PMC12796335; doi:10.1038/s41598-026-35024-4)
Supplement: Supplementary file 1 — Supplementary Material 1 [file 41598_2026_35024_MOESM1_ESM.pdf]

A Roadmap for Modeling Glass Transition Temperature

Supplementary Information for the paper
"Glass transition temperatures of pure glass-forming liquids and binary mixtures"

Vitaly Kocherbitov and Ivan Argatov

Faculty of Health and Society, Malmö University, SE-205 06 Malmö, Sweden
Biofilms – Research Centre for Biointerfaces, Malmö University, SE-205 06 Malmö, Sweden

---

Index

1 Glass transition temperature . . . 2
1.1 Equation for the glass transition temperature . . . 2
1.1.1 Activation energy, fragility, and the activation energy temperature index . . . 2
1.1.2 Arrhenius equation-based model . . . 3
1.1.3 Vogel–Fulcher–Tammann equation-based model . . . 4
1.1.4 Avramov–Milchev equation-based model . . . 4
1.1.5 Waterton–Mauro equation-based model . . . 6
1.1.6 Generalized Vogel equation-based model . . . 6
1.2 Constitutive equations in the normalized Angell coordinates . . . 7
1.2.1 Arrhenius law . . . 7
1.2.2 Avramov–Milchev model . . . 7
1.2.3 Vogel–Fulcher–Tammann (VFT) model . . . 8
1.2.4 Waterton–Mauro model . . . 8
1.2.5 Drozd-Rzoska model . . . 8
1.2.6 Cohen–Grest model . . . 9
1.2.7 Rössler model . . . 9
1.2.8 Vogel-type model . . . 9
1.3 Relation between the glass transition relaxation time and activation energy . . . 10
1.3.1 Case of the Arrhenius law . . . 10
1.3.2 Case of the Avramov–Milchev model . . . 10
1.3.3 Case of the VFT model . . . 10
1.3.4 Universal relation . . . 11
2 Glass transition mixing models . . . 11
2.1 Mixing rules . . . 12
2.1.1 Ideal dynamic mixing rules . . . 12
2.1.2 Non-ideal mixing rules . . . 12
2.2 Arrhenius equation-based mixture models . . . 12
2.2.1 Derivation of the Gordon–Taylor equation from the Arrhenius law-based model . . . 13
2.2.2 The GT equation follows from the fragility/activation energy universal relation . . . 13
2.3 Avramov–Milchev equation-based mixture models . . . 14
2.3.1 Derivation of the GT-type glass transition temperature equation . . . 14
2.3.2 Application of the non-ideal mixing rule . . . 15
2.4 Towards the Waterton–Mauro equation-based mixture model . . . 15

Keywords: Glass transition temperature, Arrhenius equation, Activation energy

---

1 Glass transition temperature

1.1 Equation for the glass transition temperature

Here we generally assume the super-Arrhenius evolution of relaxation time

$$\tau = \tau_0 \exp\left(\frac{E'_\mathrm{a}(T)}{RT}\right), \tag{SI.1}$$

and consider a number of specific models, all of which stem from the Arrhenius law.

Whereas Bartenev's approach [1], which adopts the kinetic glass transition criterion $q\tau_\mathrm{g} = \mathrm{const}$ for determining the glass transition temperature, T_g, from a corresponding characteristic relaxation time τ_g, is popular in the literature, following Vol'kenshtein and Ptitsyn [2], we define the glass transition temperature by the equation

$$\left.\frac{\mathrm{d}\tau}{\mathrm{d}T}\right|_{T=T_\mathrm{g}} = -\frac{1}{|q|}, \tag{SI.2}$$

where q is the temperature scanning rate, i.e., $q = \mathrm{d}T/\mathrm{d}t$.

1.1.1 Activation energy, fragility, and the activation energy temperature index

Let a glassformer is characterized by the constitutive equation

$$\tau = f(T), \tag{SI.3}$$

which defines the relaxation time τ as a function of the temperature T.

A characteristic relaxation time, τ_0, formally can be introduced as the following limit provided it exists:

$$\tau_0 = \lim_{T\to\infty} f(T). \tag{SI.4}$$

In what follows, we will make use of the notation

$$a = \tau_0 |q|, \tag{SI.5}$$

where q is the temperature scanning rate. We note that the parameter a has the dimension of temperature.

The activation energy, $E_\mathrm{a}(T)$, as a function of time is defined by the formula

$$\frac{E_\mathrm{a}(T)}{R} = \frac{\mathrm{d}\ln\tau}{\mathrm{d}(1/T)} \equiv b(T), \tag{SI.6}$$

where R is the molar gas constant, and $b(T)$ is the normalized activation energy.

In view of the super-Arrhenius law (SI.1), we also introduce the apparent activation energy, $E'_\mathrm{a}(T)$, as follows [3]:

$$\frac{E'_\mathrm{a}(T)}{R} = T\ln\frac{\tau(T)}{\tau_0} \equiv \mathrm{e}_\mathrm{a}(T). \tag{SI.7}$$

Here, $\mathrm{e}_\mathrm{a}(T)$ is the normalized apparent activation energy.

Following [4], we define the so-called kinetic fragility index

$$m = \left.\frac{\partial \log_{10}\tau}{\partial(T_\mathrm{g}/T)}\right|_{T=T_\mathrm{g}} = \frac{1}{\ln 10}\left(\frac{\partial\ln\tau}{\partial(T_\mathrm{g}/T)}\right)_{T=T_\mathrm{g}} = -\frac{T_\mathrm{g}}{\ln 10}\left(\frac{\partial\ln\tau}{\partial T}\right)_{T=T_\mathrm{g}}. \tag{SI.8}$$

From Eqs. (SI.6) and (SI.8), the following universal relation can be derived:

$$m = \frac{b_\mathrm{g}}{T_\mathrm{g}\ln 10}, \tag{SI.9}$$

where b_g is the normalized characteristic activation energy defined as

$$b_\mathrm{g} = b(T_\mathrm{g}). \tag{SI.10}$$

Thus, having the function $b(T)$, it is easy to evaluate the fragility m by making use of formula (SI.9).

Following [5], we also consider the Dyre–Olsen (OD) activation energy temperature index

$$I_{\mathrm{OD}}(T) = -\frac{\mathrm{d}\ln \mathrm{e_a}(T)}{\mathrm{d}\ln T}. \tag{SI.11}$$

In view of (SI.6), formula (SI.11) can be rewritten in the equivalent forms as

$$I_{\mathrm{OD}}(T) = -T\frac{\mathrm{d}\ln \mathrm{e_a}(T)}{\mathrm{d}T} = -\frac{T}{\mathrm{e_a}(T)}\frac{\mathrm{d}\mathrm{e_a}(T)}{\mathrm{d}T}. \tag{SI.12}$$

From Eqs. (SI.8) and (SI.12), in view of (SI.7), it follows that

$$\frac{1}{\ln 10}\ln\frac{\tau(T_{\mathrm{g}})}{\tau_0} = \frac{m}{I_{\mathrm{OD}}(T_{\mathrm{g}})+1}. \tag{SI.13}$$

Let us introduce the short-hand notation

$$\tau_{\mathrm{g}} = \tau(T_{\mathrm{g}}), \quad i_{\mathrm{g}} = I_{\mathrm{OD}}(T_{\mathrm{g}}). \tag{SI.14}$$

Then, Eq. (SI.15) (see also [3]) can be rewritten as

$$\lg\frac{\tau_{\mathrm{g}}}{\tau_0} = \frac{m}{i_{\mathrm{g}}+1}, \tag{SI.15}$$

where $\lg x = \log_{10} x$ is the base-10 logarithm of x.

1.1.2 Arrhenius equation-based model

The Arrhenius equation can be written in the form

$$\tau = \tau_0 \exp\left(\frac{b}{T}\right), \tag{SI.16}$$

where b is a constant, which has the meaning of the ratio of the activation energy E_{a} to the molar gas constant R. In this basic case we have $Rb = E_{\mathrm{a}} = E'_{\mathrm{a}}$ (in complete agreement with the definitions (SI.6) and (SI.7)).

The substitution of (SI.16) into (SI.2) leads to the equation

$$\frac{ab}{T_{\mathrm{g}}^2}\exp\left(\frac{b}{T_{\mathrm{g}}}\right) = 1, \tag{SI.17}$$

where we have used the notation (SI.5).

Via the substitution

$$y = \frac{b}{2T_{\mathrm{g}}}, \tag{SI.18}$$

we can transform Eq. (SI.17) to the form

$$y\exp(y) = \frac{1}{2}\sqrt{\frac{b}{a}}. \tag{SI.19}$$

Indeed, according to (SI.18), we have $T_{\mathrm{g}} = b/(2y)$. Substituting this expression for T_{g} into Eq. (SI.17), we arrive at the equation $4y^2\exp(2y) = b/a$. Now, by applying the square root to this equation, we derive the equation $2y\exp(y) = \sqrt{b/a}$, which is the same as Eq. (SI.19).

Now, Eq. (SI.19) can be solved in terms of the principal branch, $W_0(x)$, of the Lambert function that solves the transcendental equation $y\exp(y) = x$ in the form $y = W_0(x)$. In this way, we obtain

$$T_{\mathrm{g}} = \frac{b}{2W_0\left(\frac{1}{2}\sqrt{\frac{b}{a}}\right)}. \tag{SI.20}$$

Formula (SI.20) describes the dependence of the glass transition on the scanning rate in the framework of the Arrhenius equation-based model.

Remark 1. *We note that Eq.* (SI.20) *can be rewritten as*

$$T_\mathrm{g} = \frac{b}{2W_0(\theta)}, \tag{SI.21}$$

where we have introduced the notation

$$\theta = \frac{1}{2}\sqrt{\frac{b}{a}}. \tag{SI.22}$$

In view of (SI.18)*,* (SI.19)*, and* (SI.22)*, we have*

$$\theta = \frac{b}{2T_\mathrm{g}} \exp\Big(\frac{b}{2T_\mathrm{g}}\Big). \tag{SI.23}$$

Now, by taking into account the universal relation (SI.9)*, we transform Eq.* (SI.23) *to the form*

$$\theta = m\frac{\ln 10}{2} \exp\Big(m\frac{\ln 10}{2}\Big), \tag{SI.24}$$

where m is the fragility index.

1.1.3 Vogel–Fulcher–Tammann equation-based model

The Vogel–Fulcher–Tammann (VFT) equation (see, e.g., [6]) can be represented in the form

$$\tau = \tau_0 \exp\Big(\frac{B}{T - T_0}\Big), \tag{SI.25}$$

where B is a constant, and T_0 is some characteristic temperature.

The substitution of (SI.25) into (SI.2), in view of (SI.5), leads to the equation

$$\frac{aB}{(T_\mathrm{g} - T_0)^2} \exp\Big(\frac{B}{T_\mathrm{g} - T_0}\Big) = 1, \tag{SI.26}$$

whose exact solution is given by

$$T_\mathrm{g} = T_0 + \frac{B}{2W_0\Big(\frac{1}{2}\sqrt{\frac{B}{a}}\Big)}. \tag{SI.27}$$

It can be easily verified that the substitution of (SI.25) into (SI.6) yields

$$b(T) = \frac{B}{\Big(1 - \frac{T_0}{T}\Big)^2}, \tag{SI.28}$$

whereas according to Eq. (SI.7), we have

$$\mathrm{e_a}(T) = \frac{B}{1 - \frac{T_0}{T}}. \tag{SI.29}$$

1.1.4 Avramov–Milchev equation-based model

The Avramov–Milchev (AM) equation [7] can be represented in the form

$$\tau = \tau_0 \exp\Big\{\Big(\frac{\mathcal{B}}{T}\Big)^\alpha\Big\}, \tag{SI.30}$$

where $\mathcal{B}$ is a constant, and α is some dimensionless fitting parameter.

The substitution of (SI.30) into (SI.2), in view of (SI.5), results in the equation

$$\frac{\alpha a}{T_\mathrm{g}}\Big(\frac{\mathcal{B}}{T_\mathrm{g}}\Big)^\alpha \exp\Big\{\Big(\frac{\mathcal{B}}{T_\mathrm{g}}\Big)^\alpha\Big\} = 1. \tag{SI.31}$$

By making use of the Lambert function, we can resolve Eq. (SI.31) in the same way as Eq. (SI.19), so that

$$T_{\mathrm{g}} = \mathcal{B}\biggl\{\frac{(\alpha+1)}{\alpha}W_0\biggl(\frac{\alpha}{(\alpha+1)}\Bigl(\frac{\mathcal{B}}{\alpha a}\Bigr)^{\alpha/(\alpha+1)}\biggr)\biggr\}^{-1/\alpha}. \tag{SI.32}$$

Now, the substitution of (SI.30) into (SI.6) yields

$$b(T) = \alpha\mathcal{B}\Bigl(\frac{\mathcal{B}}{T}\Bigr)^{\alpha-1}, \tag{SI.33}$$

whereas according to Eq. (SI.7), we have

$$\mathrm{e_a}(T) = \mathcal{B}\Bigl(\frac{\mathcal{B}}{T}\Bigr)^{\alpha-1}. \tag{SI.34}$$

We note that for $\alpha = 1$ the AM model reduces to the Arrhenius equation-based model, and Eq. (SI.33) predicts that $b(T) \equiv \mathrm{const}$ and $\mathrm{e_a}(T) \equiv \mathrm{const}$.

We observe that, in view of (SI.33), Eq. (SI.31) can be represented as

$$\frac{ab_{\mathrm{g}}}{T_{\mathrm{g}}^2}\exp\Bigl(\frac{b_{\mathrm{g}}}{\alpha T_{\mathrm{g}}}\Bigr) = 1, \tag{SI.35}$$

where, in view of (SI.10), we have introduced the notation

$$b_{\mathrm{g}} = b(T_{\mathrm{g}}) = \alpha T_{\mathrm{g}}\Bigl(\frac{\mathcal{B}}{T_{\mathrm{g}}}\Bigr)^{\alpha}. \tag{SI.36}$$

By simple algebra, Eq. (SI.35) can be transformed to the form

$$\frac{b_{\mathrm{g}}}{2\alpha T_{\mathrm{g}}}\exp\Bigl(\frac{b_{\mathrm{g}}}{2\alpha T_{\mathrm{g}}}\Bigr) = \frac{1}{2\alpha}\sqrt{\frac{b_{\mathrm{g}}}{a}}, \tag{SI.37}$$

from where we find that

$$T_{\mathrm{g}} = \frac{b_{\mathrm{g}}}{2\alpha W_0\Bigl(\frac{1}{2\alpha}\sqrt{\frac{b_{\mathrm{g}}}{a}}\Bigr)}. \tag{SI.38}$$

We note that for $\alpha = 1$, formula (SI.38) reduces to (SI.20), since b_{g} coincides with b in the latter case.

Remark 2. *We note that Eq.* (SI.38) *can be rewritten as*

$$T_{\mathrm{g}} = \frac{b_{\mathrm{g}}}{2\alpha W_0(\theta)}. \tag{SI.39}$$

where we have introduced the notation

$$\theta = \frac{1}{2\alpha}\sqrt{\frac{b_{\mathrm{g}}}{a}}. \tag{SI.40}$$

In view of (SI.37) *and* (SI.40)*, we have*

$$\theta = \frac{b_{\mathrm{g}}}{2\alpha T_{\mathrm{g}}}\exp\Bigl(\frac{b_{\mathrm{g}}}{2\alpha T_{\mathrm{g}}}\Bigr). \tag{SI.41}$$

Now, by taking into account the universal relation (SI.9)*, we transform Eq.* (SI.41) *to the form*

$$\theta = m\frac{\ln 10}{2\alpha}\exp\Bigl(m\frac{\ln 10}{2\alpha}\Bigr), \tag{SI.42}$$

where m is the fragility index.

1.1.5 Waterton–Mauro equation-based model

The Waterton–Mauro (WM) equation [8, 9], which is also known [10] as the Mauro–Yue–Ellison–Gupta–Allan (MYEGA) equation, can be represented in the following form:

$$\tau = \tau_0 \exp\Big\{\frac{\mathfrak{B}}{T}\exp\Big(\frac{C}{T}\Big)\Big\}. \tag{SI.43}$$

Here, $\mathfrak{B}$ is a constant, and C is some parameter having the dimension of temperature.

It can be verified that the substitution of (SI.43) into (SI.6) yields

$$b(T) = \mathfrak{B}\exp\Big(\frac{C}{T}\Big)\Big[1+\frac{C}{T}\Big], \tag{SI.44}$$

whereas according to Eq. (SI.7), we have

$$\mathrm{e_a}(T) = \frac{\mathfrak{B}}{T}\exp\Big(\frac{C}{T}\Big). \tag{SI.45}$$

By substituting (SI.43) into (SI.2), in view of (SI.5), we arrive at the equation

$$\frac{a\mathfrak{B}}{T_\mathrm{g}^2}\exp\Big\{\frac{\mathfrak{B}}{T_\mathrm{g}}\exp\Big(\frac{C}{T_\mathrm{g}}\Big)\Big\}\exp\Big(\frac{C}{T_\mathrm{g}}\Big)\Big(1+\frac{C}{T_\mathrm{g}}\Big) = 1. \tag{SI.46}$$

Now, making use of the substitution

$$z = \frac{\mathfrak{B}}{2T_\mathrm{g}}\exp\Big(\frac{C}{T_\mathrm{g}}\Big), \tag{SI.47}$$

we can transform Eq. (SI.46) as follows:

$$z\exp(z) = \frac{1}{2}\sqrt{\frac{\mathfrak{B}}{a}}\Big(1+\frac{C}{T_\mathrm{g}}\Big)^{-1}. \tag{SI.48}$$

It should be emphasized that the above equation contains the unknown glass transition temperature on both sides.

Further, it is not difficult to verify that the substitution of (SI.43) into (SI.12) yields

$$I_\mathrm{OD}(T) = \frac{C}{T}. \tag{SI.49}$$

Let us introduce the short-hand notation

$$i_\mathrm{g} = \frac{C}{T_\mathrm{g}}, \tag{SI.50}$$

which is in a complete agreement with the second formula (SI.14).

Based on the analysis [5] for the inverse index $1/I(T)$, one can assume that the value of the parameter i_g does not vary much, or it can be assumed to be known *a priori*. Then, in view of (SI.47), Eq. (SI.48) can be resolved as

$$T_\mathrm{g} = \frac{\mathfrak{B}\exp(i_\mathrm{g})}{2W_0\Big(\dfrac{1}{2(1+i_\mathrm{g})}\sqrt{\dfrac{\mathfrak{B}}{a}}\Big)}. \tag{SI.51}$$

It should be underlined that, if the value of i_g is not known exactly, then formula (SI.51) gives an approximation for the glass transition temperature. In the general case, the transcendental equation (SI.46) can be solved numerically.

1.1.6 Generalized Vogel equation-based model

The Vogel-type (VFT_θ) equation (see, e.g., [11, 10]) can be represented in the form

$$\tau = \tau_0 \exp\Big\{\Big(\frac{\Phi}{(T-T_0)}\Big)^\theta\Big\}, \tag{SI.52}$$

where Φ and θ are constants, and T_0 is some characteristic temperature.

The substitution of (SI.52) into (SI.2), in view of (SI.5), leads to the equation

$$\frac{\theta a}{\Phi}\Big(\frac{\Phi}{(T-T_0)}\Big)^{\theta+1}\exp\Big\{\Big(\frac{\Phi}{(T-T_0)}\Big)^{\theta}\Big\}=1,$$

which can be further transformed to the form $y\exp(y)=x$, so that after some simple algebra we find

$$T_{\rm g}=T_0+\Phi\Big\{\frac{(\theta+1)}{\theta}W_0\Big(\frac{\theta}{\theta+1}\Big(\frac{\Phi}{\theta a}\Big)^{\theta/(\theta+1)}\Big)\Big\}^{-1/\theta}. \qquad \text{(SI.53)}$$

It can be verified that the substitution of (SI.52) into (SI.6) yields

$$b(T)=\frac{\theta\Phi^{\theta}T^2}{(T-T_0)^{\theta+1}}, \qquad \text{(SI.54)}$$

whereas according to Eq. (SI.7), we have

$$\mathrm{e_a}(T)=\frac{\Phi^{\theta}T}{(T-T_0)^{\theta}}. \qquad \text{(SI.55)}$$

1.2 Constitutive equations in the normalized Angell coordinates

In view of (SI.4), the general constitutive equation (SI.3) can be rewritten in the form

$$\tau=\tau_0\bar{f}(T), \qquad \text{(SI.56)}$$

where $\bar{f}(T)$ is a dimensionless function of the temperature T such that $\bar{f}(T)\to 1$ as $T\to\infty$.

In what follows, we make use of the notation

$$\tau_{\rm g}=\tau_0\bar{f}(T_{\rm g}), \qquad \text{(SI.57)}$$

where $T_{\rm g}$ is the glass transition temperature.

In the Angell coordinates, the relaxation time/temperature relation is represented as a functional dependence of the ratio $\ln(\tau/\tau_0)/\ln(\tau_{\rm g}/\tau_0)$ as a function of the ratio $T_{\rm g}/T$.

To simplify the formulas, we put

$$y=\frac{\ln(\tau/\tau_0)}{\ln(\tau_{\rm g}/\tau_0)},\quad x=\frac{T_{\rm g}}{T}. \qquad \text{(SI.58)}$$

Thus, the constitutive equation in the Angell coordinates is given by the function $y(x)$.

1.2.1 Arrhenius law

In view of (SI.58), the Arrhenius equation (SI.16) can be recast in the form

$$y=x, \qquad \text{(SI.59)}$$

which does not contain free parameters.

1.2.2 Avramov–Milchev model

From Eq. (SI.30), we find

$$\ln(\tau/\tau_0)=\Big(\frac{\mathcal{B}}{T}\Big)^{\alpha},\quad \ln(\tau_{\rm g}/\tau_0)=\Big(\frac{\mathcal{B}}{T_{\rm g}}\Big)^{\alpha},$$

from where it follows the Avramov–Milchev model in the Angell coordinates

$$y=x^{\alpha}. \qquad \text{(SI.60)}$$

1.2.3 Vogel–Fulcher–Tammann (VFT) model

From Eq. (SI.25), we readily find that

$$\ln(\tau/\tau_0) = \frac{B}{T - T_0}, \quad \ln(\tau_g/\tau_0) = \frac{B}{T_g - T_0},$$

so that

$$\frac{\ln(\tau/\tau_0)}{\ln(\tau_g/\tau_0)} = \frac{1 - (T_0/T_g)}{(T/T_g) - (T_0/T_g)}. \tag{SI.61}$$

Let us introduce the notation

$$x_0 = \frac{T_g}{T_0}. \tag{SI.62}$$

Then, in view of (SI.58) and (SI.62), Eq. (SI.61) can be rewritten in the one-parameter form as

$$y = \frac{x(x_0 - 1)}{x_0 - x}, \tag{SI.63}$$

where it is assumed that $x_0 > 1$.

1.2.4 Waterton–Mauro model

In the same way as before but now using Eq. (SI.43), we obtain

$$\ln(\tau/\tau_0) = \frac{\mathfrak{B}}{T}\exp\Big(\frac{C}{T}\Big), \quad \ln(\tau_g/\tau_0) = \frac{\mathfrak{B}}{T_g}\exp\Big(\frac{C}{T_g}\Big),$$

and, respectively, arrive at the equation

$$\frac{\ln(\tau/\tau_0)}{\ln(\tau_g/\tau_0)} = \frac{T_g}{T}\exp\Big(\frac{C}{T} - \frac{C}{T_g}\Big). \tag{SI.64}$$

Let us introduce the notation

$$c = \frac{C}{T_g}. \tag{SI.65}$$

Then, in view of (SI.58) and (SI.65), Eq. (SI.64) can be transformed to the form

$$y = x\exp\{-c(1 - x)\}. \tag{SI.66}$$

1.2.5 Drozd-Rzoska model

The Drozd-Rzoska equation [12] has the form

$$\tau = C_\Omega\left(\frac{T - T_g^*}{T}\right)^{-\Omega}\exp\left(\Omega\frac{(T - T_g^*)}{T}\right), \tag{SI.67}$$

where C_Ω, T_g^*, and Ω are constant parameters.

It can be easily verified that

$$\tau_0 = \lim_{T\to\infty}\tau(T) = C_\Omega\exp(\Omega). \tag{SI.68}$$

In view of (SI.68), Eq. (SI.67) can be simplified as

$$\tau = \tau_0\left(\frac{T}{T - T_g^*}\right)^{\Omega}\exp\left(-\Omega\frac{T_g^*}{T}\right). \tag{SI.69}$$

From Eq. (SI.69), it follows that

$$\ln(\tau/\tau_0) = \Omega\ln\frac{T}{T - T_g^*} - \Omega\frac{T_g^*}{T}, \quad \ln(\tau_g/\tau_0) = \Omega\ln\frac{T_g}{T_g - T_g^*} - \Omega\frac{T_g^*}{T_g}. \tag{SI.70}$$

Let us introduce the notation

$$\gamma_* = \frac{T_g^*}{T_g}. \tag{SI.71}$$

Then, in view of (SI.58) and (SI.71), the Drozd-Rzoska model in the Angell coordinates can be represented as

$$y = \frac{\gamma_* x + \ln(1 - \gamma_* x)}{\gamma_* + \ln(1 - \gamma_*)}. \tag{SI.72}$$

1.2.6 Cohen–Grest model

By using a free-volume approach, Cohen and Grest have derived the following four-parameter model [13] (cf. formula (48) for the viscosity in their paper):

$$\ln\tau(T) = A + \frac{B}{T - T_0 + \sqrt{(T-T_0)^2 + C}}, \tag{SI.73}$$

where T_0, A, B, and C are constant parameters.

Using the obvious parameter change $A = \ln\tau_0$ in view of (SI.4), formula (SI.73) can be rewritten in the already familiar form

$$\tau = \tau_0 \exp\biggl(\frac{B}{T - T_0 + \sqrt{(T-T_0)^2 + C}}\biggr). \tag{SI.74}$$

From Eq. (SI.74), after some simple algebra it follows that

$$\ln(\tau/\tau_0) = \frac{B}{C}\bigl\{\sqrt{(T-T_0)^2 + C} - (T - T_0)\bigr\}, \quad \ln(\tau_{\rm g}/\tau_0) = \frac{B}{C}\bigl\{\sqrt{(T_{\rm g}-T_0)^2 + C} - (T_{\rm g} - T_0)\bigr\}. \tag{SI.75}$$

Let us introduce the notation

$$x_0 = \frac{T_{\rm g}}{T_0}, \quad \gamma_0 = \frac{C}{T_0^2}. \tag{SI.76}$$

Then, in view of (SI.58) and (SI.76), the Cohen–Grest model (SI.74) in the Angell coordinates can be represented as

$$y = \frac{\sqrt{(x_0 - x)^2 + \gamma_0 x^2} - (x_0 - x)}{\sqrt{(x_0-1)^2 + \gamma_0} - (x_0 - 1)}. \tag{SI.77}$$

1.2.7 Rössler model

The Rössler model (see [14, 10]) can be written in the form

$$\tau = \tau_0 \exp\biggl\{\frac{E_0}{T}\biggl[1 + a\exp\biggl(-\lambda\Bigl(\frac{T}{T_{\rm A}} - 1\Bigr)\biggr)\biggr]\biggr\}. \tag{SI.78}$$

where $T_{\rm A}$ is the crossover temperature from the super-Arrhenius to Arrhenius behavior in high-temperature regions, E_0 is the high-temperature activation energy, and a and λ are constant parameters.

Equation (SI.78) implies that

$$\ln(\tau/\tau_0) = \frac{E_0}{T}\biggl[1 + a\exp\biggl(-\lambda\Bigl(\frac{T}{T_{\rm A}} - 1\Bigr)\biggr)\biggr], \quad \ln(\tau_{\rm g}/\tau_0) = \frac{E_0}{T_{\rm g}}\biggl[1 + a\exp\biggl(-\lambda\Bigl(\frac{T_{\rm g}}{T_{\rm A}} - 1\Bigr)\biggr)\biggr]. \tag{SI.79}$$

Let us introduce the notation

$$\tilde{a} = a\exp(\lambda), \quad x_{\rm A} = \frac{T_{\rm g}}{T_{\rm A}}. \tag{SI.80}$$

Then, in view of (SI.58) and (SI.80), the Rössler model (SI.78) in the Angell coordinates can be represented as

$$y = x\frac{\bigl[1 + \tilde{a}\exp(-\lambda x_{\rm A}/x)\bigr]}{1 + \tilde{a}\exp(-\lambda x_{\rm A})}, \tag{SI.81}$$

where $x_{\rm A}$, $\tilde{a}$, and λ are dimensionless parameters.

1.2.8 Vogel-type model

From Eq. (SI.52), we derive

$$\ln(\tau/\tau_0) = \Bigl(\frac{\Phi}{(T-T_0)}\Bigr)^{\theta}, \quad \ln(\tau_{\rm g}/\tau_0) = \Bigl(\frac{\Phi}{(T_{\rm g}-T_0)}\Bigr)^{\theta},$$

so that

$$\frac{\ln(\tau/\tau_0)}{\ln(\tau_{\rm g}/\tau_0)} = \frac{\bigl(1 - (T_0/T_{\rm g})\bigr)^{\theta}}{\bigl[(T/T_{\rm g}) - (T_0/T_{\rm g})\bigr]^{\theta}}. \tag{SI.82}$$

Then, in view of (SI.58) and (SI.62), the VFT$_\theta$ model (SI.82) can be rewritten as

$$y = \frac{x^{\theta}(x_0 - 1)^{\theta}}{(x_0 - x)^{\theta}}. \tag{SI.83}$$

1.3 Relation between the glass transition relaxation time and activation energy

1.3.1 Case of the Arrhenius law

By the definition of the glass transition relaxation time (see formula (SI.57)), in the case of the Arrhenius equation (SI.16) we have

$$\ln(\tau_g/\tau_0) = \frac{b}{T_g}, \tag{SI.84}$$

from where, in view of the solution for the glass transition temperature (SI.20), we derive the relation

$$\ln(\tau_g/\tau_0) = 2W_0\left(\frac{1}{2}\sqrt{\frac{b}{a}}\right). \tag{SI.85}$$

Now, by the definition of the Lambert function $W_0(x)$, we can solve the equation $W_0(x) = y$ in the form $y\exp(y) = x$. In this way, Eq. (SI.85) yields

$$\frac{b}{a} = \frac{\tau_g}{\tau_0}\ln^2\left(\frac{\tau_g}{\tau_0}\right). \tag{SI.86}$$

In view of (SI.5), relation (SI.86) can be also recast in the form

$$b = |q|\tau_g\ln^2\left(\frac{\tau_g}{\tau_0}\right), \tag{SI.87}$$

where q is the temperature scanning rate.

1.3.2 Case of the Avramov–Milchev model

From Eq. (SI.30), we readily get

$$\ln\left(\frac{\tau_g}{\tau_0}\right) = \left(\frac{\mathcal{B}}{T_g}\right)^{\alpha}. \tag{SI.88}$$

On the other hand, according to (SI.38), we have

$$\left(\frac{\mathcal{B}}{T_g}\right)^{\alpha} = \frac{b_g}{\alpha T_g}, \tag{SI.89}$$

where b_g is the glass transition activation energy.

Now, taking into account the solution for the glass transition temperature (SI.38), we can write

$$\frac{b_g}{T_g} = 2\alpha W_0\left(\frac{1}{2\alpha}\sqrt{\frac{b_g}{a}}\right). \tag{SI.90}$$

Thus, collecting formulas (SI.88)–(SI.90), we arrive at the relation

$$\ln(\tau_g/\tau_0) = 2W_0\left(\frac{1}{2\alpha}\sqrt{\frac{b_g}{a}}\right). \tag{SI.91}$$

Now, by the definition of the Lambert function, we invert Eq. (SI.91) and get

$$\frac{b_g}{a} = \alpha^2\frac{\tau_g}{\tau_0}\ln^2\left(\frac{\tau_g}{\tau_0}\right). \tag{SI.92}$$

We observe that Eq. (SI.92) relates the glass transition activation energy b_g to the glass transition relaxation time τ_g in the framework of the Avramov–Milchev model.

1.3.3 Case of the VFT model

From Eq. (SI.25), we readily find that

$$\ln\left(\frac{\tau_g}{\tau_0}\right) = \frac{B}{T_g - T_0}. \tag{SI.93}$$

So, in view of (SI.25), we have

$$\ln\left(\frac{\tau_g}{\tau_0}\right) = 2W_0\left(\frac{1}{2}\sqrt{\frac{B}{a}}\right), \tag{SI.94}$$

from where it follows that

$$\frac{B}{a} = \frac{\tau_g}{\tau_0}\ln^2\left(\frac{\tau_g}{\tau_0}\right). \tag{SI.95}$$

Finally, we recall that according to (SI.28) we have

$$b_g = \frac{BT_g^2}{(T_g - T_0)^2}. \tag{SI.96}$$

By using Eqs. (SI.93) and (SI.96), we can exclude the parameter B and resolve the obtained quadratic algebraic equation for the glass transition temperature

$$T_g = \frac{b_g + \sqrt{b_g^2 - 4b_g T_0 \ln(\tau_g/\tau_0)}}{2\ln(\tau_g/\tau_0)}. \tag{SI.97}$$

In view of (SI.96) and (SI.97), Eq. (SI.96) can be further transformed to an implicit functional algebraic relation between b_g and τ_g.

1.3.4 Universal relation

We recall that by the definition (SI.6) we have

$$b(T) = \frac{1}{\tau(T)}\frac{d\tau(T)}{d(1/T)}, \tag{SI.98}$$

so that

$$b(T) = -\frac{T^2}{\tau(T)}\frac{d\tau(T)}{dT}. \tag{SI.99}$$

Thus, in view of (SI.10) and (SI.14), the substitution of $T = T_g$ into Eq. (SI.99) yields

$$b_g = -\frac{T_g^2}{\tau_g}\left(\frac{d\tau(T)}{dT}\right)_{T=T_g}. \tag{SI.100}$$

Now, in light of (SI.2), we can rewrite formula (SI.100) as

$$b_g = \frac{T_g^2}{\tau_g|q|}. \tag{SI.101}$$

Finally, by rearranging Eq. (SI.101), we obtain the relation

$$\tau_g = \frac{T_g^2}{b_g|q|}, \tag{SI.102}$$

which is universal in a sense that it does not depend on the specific form of the super-Arrhenius law (SI.1).

2 Glass transition mixing models

Observe that the general constitutive equation (SI.3), when rewritten in the form (SI.56), contains one parameter with the physical dimension of time (denoted by τ_0 and replaced with the dimensionless parameter a, using the relation $a = q\tau_0$) and one parameter with the dimension of temperature. This parameter, in the case of the Arrhenius equation (SI.16), is denoted by b. In the case of the Avramov–Milchev equation-based model (SI.30), using the formula (SI.36), we can introduce an analogous parameter (denoted by b_g). In the latter case, the constitutive equation also includes an additional dimensionless parameter, α.

2.1 Mixing rules

2.1.1 Ideal dynamic mixing rules

Analogously to the Arrhenius mixing rule for viscosity $\ln\eta = w_1 \ln\eta_1 + w_2 \ln\eta_2$ we formulate the ideal dynamic mixing rules as follows:

$$b = w_1 b_1 + w_2 b_2, \tag{SI.103}$$

$$\ln\frac{a}{b} = w_1 \ln\frac{a_1}{b_1} + w_2 \ln\frac{a_2}{b_2}, \tag{SI.104}$$

where w_1 and w_2 are the weight fractions of the components, which satisfy the normalization relation

$$w_1 + w_2 = 1. \tag{SI.105}$$

The introduction of any additional dimensionless parameter (such as α) will require formulating the corresponding mixing rule.

2.1.2 Non-ideal mixing rules

In light of (SI.105), Eq. (SI.103) can be rewritten in the equivalent form

$$b = b_1 + (b_2 - b_1)w_2.$$

The above linear relation can be generalized as

$$b = b_1 + (b_2 - b_1)w_2^n, \tag{SI.106}$$

where n is a positive dimensionless parameter.

It is obvious that different non-ideal mixing rules can be designed. For instance, by introducing two additional fitting parameters, formula (SI.103) can be straightforwardly generalized as follows: $b = b_1 w_1^{n_1} + b_2 w_2^{n_2}$.

2.2 Arrhenius equation-based mixture models

According to the Lambert function-involved representation (SI.20), we have

$$T_{g1} = \frac{b_1}{2W_0\left(\dfrac{1}{2}\sqrt{\dfrac{b_1}{a_1}}\right)}, \qquad T_{g2} = \frac{b_2}{2W_0\left(\dfrac{1}{2}\sqrt{\dfrac{b_2}{a_2}}\right)}. \tag{SI.107}$$

Assuming that the same type of relation holds for the mixture, we can write:

$$T_g = \frac{b}{2W_0\left(\dfrac{1}{2}\sqrt{\dfrac{b}{a}}\right)}. \tag{SI.108}$$

The application of the first ideal dynamic mixing rule (SI.103), in view of (SI.107), yields

$$T_g = \frac{w_1 T_{g1} W_0\left(\dfrac{1}{2}\sqrt{\dfrac{b_1}{a_1}}\right) + w_2 T_{g2} W_0\left(\dfrac{1}{2}\sqrt{\dfrac{b_2}{a_2}}\right)}{W_0\left(\dfrac{1}{2}\sqrt{\dfrac{b}{a}}\right)}. \tag{SI.109}$$

Now, applying the second ideal dynamic mixing rule (SI.104), we obtain

$$\ln\left[\left(\frac{b_1}{a_1}\right)^{w_1}\left(\frac{b_2}{a_2}\right)^{w_2}\right] = \ln\left(\frac{b}{a}\right),$$

from where it immediately follows that

$$\frac{b}{a} = \left(\frac{b_1}{a_1}\right)^{w_1}\left(\frac{b_2}{a_2}\right)^{w_2}. \tag{SI.110}$$

Finally, the substitution of (SI.110) into the denominator in (SI.109) results in the formula

$$T_g = \frac{w_1 T_{g1} W_0\left(\dfrac{1}{2}\sqrt{\dfrac{b_1}{a_1}}\right) + w_2 T_{g2} W_0\left(\dfrac{1}{2}\sqrt{\dfrac{b_2}{a_2}}\right)}{W_0\left(\dfrac{1}{2}\sqrt{\left(\dfrac{b_1}{a_1}\right)^{w_1}\left(\dfrac{b_2}{a_2}\right)^{w_2}}\right)}. \tag{SI.111}$$

2.2.1 Derivation of the Gordon–Taylor equation from the Arrhenius law-based model

It is well known that for large values of the argument, the principal branch of the Lambert W function has the asymptotic expansion

$$W_0(x) = \ln x - \ln\ln x + o(1), \quad x \to \infty, \tag{SI.112}$$

where the term $o(1)$ vanishes as x tends to infinity.

Keeping only the first term of the expansion (SI.112), we obtain the simple approximation $W_0(x) \approx \ln x$ for large values of x, which can be represented as

$$W_0(x) \sim \ln x, \quad x \to \infty, \tag{SI.113}$$

meaning that the limit of the ratio $W_0(x)/\ln x$ is equal to 1 as x tends to infinity.

Now, in view of (SI.105), we can rewrite formula (SI.111) as

$$T_{\rm g} = \frac{w_1 T_{\rm g1} W_0\left(\frac{1}{2}\sqrt{\frac{b_1}{a_1}}\right) + w_2 T_{\rm g2} W_0\left(\frac{1}{2}\sqrt{\frac{b_2}{a_2}}\right)}{W_0\left(\frac{1}{2^{w_1+w_2}}\left(\sqrt{\frac{b_1}{a_1}}\right)^{w_1}\left(\sqrt{\frac{b_2}{a_2}}\right)^{w_2}\right)}. \tag{SI.114}$$

By using the asymptotic approximation (SI.113), we replace formula (SI.114) with the following simple approximation:

$$T_{\rm g} = \frac{w_1 T_{\rm g1} \ln\left(\frac{1}{2}\sqrt{\frac{b_1}{a_1}}\right) + w_2 T_{\rm g2} \ln\left(\frac{1}{2}\sqrt{\frac{b_2}{a_2}}\right)}{w_1 \ln\left(\frac{1}{2}\sqrt{\frac{b_1}{a_1}}\right) + w_2 \ln\left(\frac{1}{2}\sqrt{\frac{b_2}{a_2}}\right)}. \tag{SI.115}$$

Finally, formula (SI.115) can be recast in the form of the Gordon–Taylor (GT) equation

$$T_{\rm g} = \frac{w_1 T_{\rm g1} + \kappa w_2 T_{\rm g2}}{w_1 + \kappa w_2}, \tag{SI.116}$$

where

$$\kappa = \frac{\ln\left(\frac{1}{2}\sqrt{\frac{b_2}{a_2}}\right)}{\ln\left(\frac{1}{2}\sqrt{\frac{b_1}{a_1}}\right)} \simeq \frac{W_0\left(\frac{1}{2}\sqrt{\frac{b_2}{a_2}}\right)}{W_0\left(\frac{1}{2}\sqrt{\frac{b_1}{a_1}}\right)} = \frac{b_2 T_{\rm g1}}{b_1 T_{\rm g2}}. \tag{SI.117}$$

We recall that formula (SI.115) was derived from (SI.114) under the assumption that the asymptotic approximation (SI.113) holds, and this explains the second relation in (SI.123), whereas the last relation is obtained using formulas (SI.107).

2.2.2 The GT equation follows from the fragility/activation energy universal relation

In view of (SI.9), we can write

$$T_{\rm g} = \frac{b_{\rm g}}{m \ln 10}, \tag{SI.118}$$

for the mixture, and

$$T_{\rm g1} = \frac{b_{\rm g1}}{m_1 \ln 10}, \quad T_{\rm g2} = \frac{b_{\rm g2}}{m_2 \ln 10}, \tag{SI.119}$$

for the two individual components.

By using the ideal mixing rule (SI.103) for the normalized characteristic activation energy and the analogous relation for the kinetic fragility index, i.e.,

$$m = w_1 m_1 + w_2 m_2, \tag{SI.120}$$

formula (SI.118) can be recast in the form

$$T_{\rm g} = \frac{b_{\rm g1} w_1 + b_{\rm g2} w_2}{(w_1 m_1 + w_2 m_2)\ln 10}. \tag{SI.121}$$

Now, taking into account relations (SI.119), we rewrite formula (SI.121) as

$$T_{\rm g} = \frac{T_{\rm g1}m_1w_1 + T_{\rm g2}m_2w_2}{w_1m_1 + w_2m_2},$$

from where it immediately follows that

$$T_{\rm g} = \frac{w_1T_{\rm g1} + \kappa w_2T_{\rm g2}}{w_1 + \kappa w_2}, \tag{SI.122}$$

where

$$\kappa = \frac{m_2}{m_1} = \frac{b_{\rm g2}T_{\rm g1}}{b_{\rm g1}T_{\rm g2}}. \tag{SI.123}$$

2.3 Avramov–Milchev equation-based mixture models

According to formula (SI.38), we can write

$$T_{\rm g1} = \frac{b_{\rm g1}}{2\alpha_1 W_0\left(\dfrac{1}{2\alpha_1}\sqrt{\dfrac{b_{\rm g1}}{a_1}}\right)}, \qquad T_{\rm g2} = \frac{b_{\rm g2}}{2\alpha_2 W_0\left(\dfrac{1}{2\alpha_2}\sqrt{\dfrac{b_{\rm g2}}{a_2}}\right)}, \tag{SI.124}$$

and assume that

$$T_{\rm g} = \frac{b_{\rm g}}{2\alpha W_0\left(\dfrac{1}{2\alpha}\sqrt{\dfrac{b_{\rm g}}{a}}\right)}, \tag{SI.125}$$

where, according to the ideal dynamic mixing rules (SI.103) and (SI.103), we have

$$b_{\rm g} = w_1b_{\rm g1} + w_2b_{\rm g2}, \tag{SI.126}$$

$$\frac{b_{\rm g}}{a} = \left(\frac{b_{\rm g1}}{a_1}\right)^{w_1}\left(\frac{b_{\rm g2}}{a_2}\right)^{w_2}. \tag{SI.127}$$

Thus, in view of (SI.126) and (SI.127), formula (SI.125) can be transformed to the form

$$T_{\rm g} = \frac{w_1T_{\rm g1}\alpha_1W_0\left(\dfrac{1}{2\alpha_1}\sqrt{\dfrac{b_{\rm g1}}{a_1}}\right) + w_2T_{\rm g2}\alpha_2W_0\left(\dfrac{1}{2\alpha_2}\sqrt{\dfrac{b_{\rm g2}}{a_2}}\right)}{\alpha W_0\left(\dfrac{1}{2\alpha}\sqrt{\left(\dfrac{b_{\rm g1}}{a_1}\right)^{w_1}\left(\dfrac{b_{\rm g2}}{a_2}\right)^{w_2}}\right)}. \tag{SI.128}$$

Finally, the simple rule of mixtures, for example, given by $\alpha = w_1\alpha_1 + w_2\alpha_2$, can be adopted as a first approximation.

2.3.1 Derivation of the GT-type glass transition temperature equation

In view of (SI.105), we can rewrite formula (SI.128) as

$$T_{\rm g} = \frac{w_1T_{\rm g1}\alpha_1W_0\left(\dfrac{1}{2\alpha_1}\sqrt{\dfrac{b_{\rm g1}}{a_1}}\right) + w_2T_{\rm g2}\alpha_2W_0\left(\dfrac{1}{2\alpha_2}\sqrt{\dfrac{b_{\rm g2}}{a_2}}\right)}{\alpha W_0\left(\dfrac{\alpha_1^{w_1}\alpha_2^{w_2}}{\alpha}\left(\dfrac{1}{2\alpha_1}\sqrt{\dfrac{b_1}{a_1}}\right)^{w_1}\left(\dfrac{1}{2\alpha_1}\sqrt{\dfrac{b_2}{a_2}}\right)^{w_2}\right)}. \tag{SI.129}$$

By using the asymptotic approximation (SI.113), we replace formula (SI.129) with the approximate relation

$$T_{\rm g} = \frac{w_1T_{\rm g1}\alpha_1\ln\left(\dfrac{1}{2\alpha_1}\sqrt{\dfrac{b_1}{a_1}}\right) + w_2T_{\rm g2}\alpha_2\ln\left(\dfrac{1}{2\alpha_2}\sqrt{\dfrac{b_2}{a_2}}\right)}{\alpha\left\{\ln\left(\dfrac{\alpha_1^{w_1}\alpha_2^{w_2}}{\alpha}\right) + w_1\ln\left(\dfrac{1}{2\alpha_1}\sqrt{\dfrac{b_1}{a_1}}\right) + w_2\ln\left(\dfrac{1}{2\alpha_2}\sqrt{\dfrac{b_2}{a_2}}\right)\right\}}. \tag{SI.130}$$

Formula (SI.130) can be transformed to the form similar to that of the Gordon–Taylor equation (SI.116), provided the following mixing rule holds:

$$\alpha = \alpha_1^{w_1}\alpha_2^{w_2}. \tag{SI.131}$$

Then, in view of (SI.131), Eq. (SI.130) simplifies as

$$T_{\rm g} = \frac{w_1 T_{\rm g1} + \kappa w_2 T_{\rm g2}}{\dfrac{\alpha}{\alpha_1} w_1 + \dfrac{\alpha}{\alpha_2}\kappa w_2}, \tag{SI.132}$$

where, in view of (SI.113) and (SI.124), we have

$$\kappa = \frac{\alpha_2 \ln\left(\dfrac{1}{2\alpha_2}\sqrt{\dfrac{b_2}{a_2}}\right)}{\alpha_1 \ln\left(\dfrac{1}{2\alpha_1}\sqrt{\dfrac{b_1}{a_1}}\right)} \simeq \frac{\alpha_2 W_0\left(\dfrac{1}{2\alpha_2}\sqrt{\dfrac{b_2}{a_2}}\right)}{\alpha_1 W_0\left(\dfrac{1}{2\alpha_1}\sqrt{\dfrac{b_1}{a_1}}\right)} = \frac{b_2 T_{\rm g1}}{b_1 T_{\rm g2}}. \tag{SI.133}$$

Finally, taking into account relations (SI.105) and (SI.131), we rewrite formula (SI.132) in the form

$$T_{\rm g} = \frac{w_1 T_{\rm g1} + \kappa w_2 T_{\rm g2}}{\left(\dfrac{\alpha_2}{\alpha_1}\right)^{w_2} w_1 + \left(\dfrac{\alpha_1}{\alpha_2}\right)^{w_1}\kappa w_2}. \tag{SI.134}$$

2.3.2 Application of the non-ideal mixing rule

First, according to Eq. (SI.106), we have

$$b_{\rm g} = (1 - w_2^n) b_{\rm g1} + w_{\rm g2}^n b_2. \tag{SI.135}$$

Then, in view of (SI.124), (SI.125), and (SI.135), we can derive

$$T_{\rm g} = \frac{(1 - w_2^n)\alpha_1 W_0\left(\dfrac{1}{2\alpha_1}\sqrt{\dfrac{b_{\rm g1}}{a_1}}\right) T_{\rm g1} + w_{\rm g2}^n \alpha_2 W_0\left(\dfrac{1}{2\alpha_1}\sqrt{\dfrac{b_{\rm g2}}{a_2}}\right) T_{\rm g2}}{\alpha W_0\left(\dfrac{1}{2\alpha}\sqrt{\dfrac{(1 - w_2^n) b_{\rm g1} + w_{\rm g2}^n b_2}{a}}\right)}. \tag{SI.136}$$

It should be noted that, in addition to the individual component parameters a_1, α_1, a_2, and α_2, the formula (SI.136) also includes the mixture parameters a and α, which can be eliminated by applying other mixing rules.

2.4 Towards the Waterton–Mauro equation-based mixture model

According to formula (SI.44), let us introduce the notation

$$b_{\rm g} = b(T_{\rm g}) = \mathfrak{B} \exp\left(\frac{C}{T_{\rm g}}\right)\left[1 + \frac{C}{T_{\rm g}}\right], \tag{SI.137}$$

which, in view of (SI.50), can be recast as

$$b_{\rm g} = \mathfrak{B} \exp(i_{\rm g})[1 + i_{\rm g}]. \tag{SI.138}$$

Then, the glass transition temperature equation (SI.51) can be represented in the form

$$T_{\rm g} = \frac{b_{\rm g}}{2(1 + i_{\rm g}) W_0\left(\dfrac{1}{2(1 + i_{\rm g})}\sqrt{\dfrac{b_{\rm g}}{\exp(i_{\rm g})(1 + i_{\rm g}) a}}\right)}. \tag{SI.139}$$

Yet, by introducing auxiliary notation

$$a_{\rm g} = a \exp(i_{\rm g})[1 + i_{\rm g}], \tag{SI.140}$$

we can simplify the expression on the right-hand side of (SI.139) as

$$T_{\rm g} = \frac{b_{\rm g}}{2(1 + i_{\rm g}) W_0\left(\dfrac{1}{2(1 + i_{\rm g})}\sqrt{\dfrac{b_{\rm g}}{a_{\rm g}}}\right)}. \tag{SI.141}$$

Observe that formula (SI.141) as the same structure as (SI.125), which open a way for deriving a mixture model based on the Waterton–Mauro equation.

Conspectus librorum

[1] Sanditov DS, Ojovan MI. Relaxation aspects of the liquid–glass transition. Physics-Uspekhi. 2019;62(2):111-30.

[2] Vol'kenshtein MV, Ptitsyn OB. The relaxation theory of glass transition. Doklady Physics. 1955;103(5):795-8.

[3] Martinez-Garcia JC, Rzoska SJ, Drozd-Rzoska A, Martinez-Garcia J. A universal description of ultraslow glass dynamics. Nature Communications. 2013;4(1):1823.

[4] Angell CA. Formation of glasses from liquids and biopolymers. Science. 1995;267(5206):1924-35.

[5] Martinez-Garcia JC, Rzoska SJ, Drzozd-Rzoska A, Martinez-Garcia J, Mauro JC. Divergent dynamics and the Kauzmann temperature in glass forming systems. Scientific Reports. 2014;4(1):5160.

[6] Kivelson SA, Tarjus G. In search of a theory of supercooled liquids. Nature Materials. 2008;7(11):831-3.

[7] Avramov I, Milchev A. Effect of disorder on diffusion and viscosity in condensed systems. Journal of Non-Crystalline Solids. 1988;104(2-3):253-60.

[8] Mauro JC, Yue Y, Ellison AJ, Gupta PK, Allan DC. Viscosity of glass-forming liquids. Proceedings of the National Academy of Sciences. 2009;106(47):19780-4.

[9] Martinez-Garcia JC, Martinez-Garcia J, Rzoska SJ, Hulliger J. The new insight into dynamic crossover in glass forming liquids from the apparent enthalpy analysis. Journal of Chemical Physics. 2012;137(6).

[10] Drozd-Rzoska A, Rzoska SJ, Starzonek S. New scaling paradigm for dynamics in glass-forming systems. Progress in Materials Science. 2023;134:101074.

[11] Bendler JT, Shlesinger MF. Generalized Vogel law for glass-forming liquids. Journal of Statistical Physics. 1988;53:531-41.

[12] Drozd-Rzoska A. Universal behavior of the apparent fragility in ultraslow glass forming systems. Scientific Reports. 2019;9(1):6816.

[13] Cohen MH, Grest GS. Liquid-glass transition, a free-volume approach. Physical Review B. 1979;20(3):1077.

[14] Schmidtke B, Petzold N, Kahlau R, Hofmann M, Rössler EA. From boiling point to glass transition temperature: Transport coefficients in molecular liquids follow three-parameter scaling. Physical Review E—Statistical, Nonlinear, and Soft Matter Physics. 2012;86(4):041507.
